# The fertility of internal migrants to Kinshasa

**DOI:** 10.1186/s41118-017-0020-8

**Published:** 2017-05-30

**Authors:** Philip Anglewicz, Jamaica Corker, Patrick Kayembe

**Affiliations:** 1 Department of Global Community Health and Behavioral Science, School of Public Health and Tropical Medicine, Tulane University, 1440 Canal St Suite 2210, New Orleans, LA USA; 2 Bill and Melinda Gates Foundation, 440 5th Ave N., Seattle, WA 98109 USA; 3 Division of Epidemiology and Biostatistics School of Public Health, Kinshasa School of Public Health, University of Kinshasa, BP 11850 Kinshasa 1, Kinshasa, Democratic Republic of Congo

**Keywords:** Fertility, Urbanization, Internal migration, Democratic Republic of Congo

## Abstract

The rapid population growth of many African cities has important implications for population health, yet little is known about factors contributing to increasing population, such as the fertility of internal migrants. We examine whether in-migrants to Kinshasa have different fertility patterns than lifetime Kinshasa residents, and identify characteristics of migrants that may explain differences in fertility. We also use detailed migration histories to examine whether fertility differs by features of migration. We use representative data from the PMA2020 Project for 2197 women in Kinshasa, including 340 women who moved to Kinshasa. We examine differences between migrants and non-migrants in fertility and other fertility-related characteristics. We also examine whether fertility differs by duration of residence in Kinshasa, number of lifetime moves, age at first migration, urban/rural classification of birthplace, and the distinction between intra-Kinshasa migration and migration to Kinshasa.. Migrants have significantly higher fertility than permanent Kinshasa residents, but the difference is relatively small in magnitude. This higher fertility appears due in part to patterns of contraceptive use among migrants. There is noteworthy heterogeneity among migrants: higher fertility among migrants is associated with longer duration in Kinshasa, more lifetime moves, urban-Kinshasa migration, older age at first migration, and moving to Kinshasa from outside (as opposed to intra-Kinshasa migration).

## Introduction

The rapid urbanization of sub-Saharan Africa (SSA) has important implications for health in the region. The crowding and unsanitary conditions in SSA cities facilitate the proliferation of infectious and vector-borne diseases (Githeko et al. 2000; Johnson et al. 2000; Wilson 1995). Although access to health services in cities is typically better than rural areas in SSA, access is still limited to some, particularly the economically disadvantaged (Matthews et al. 2010). Urban population growth puts pressure on local governments to take care of increasing number of people, which strains the already-limited resources in developing countries (White and Lindstrom 2005). Despite the health implications, little is known about factors contributing to increasing urban population, such as the fertility of internal migrants to cities in sub-Saharan Africa.

The capital of the Democratic Republic of the Congo (DRC), Kinshasa, is one of the world’s “megacities.” With a population of over 11 million, Kinshasa is Africa's third largest city (after Lagos and Cairo) and one of the continent’s most rapidly growing urban areas (United Nations 2015a). By 2020, Kinshasa is projected to add four million new inhabitants, making it Africa’s fastest-growing city (UN-Habitat 2010). As with other countries in the region, despite the considerable increase in the population size of Kinshasa, little is known about the factors contributing to population growth in this context (Piermay 1997; Corker 2015).

The relatively small body of research on fertility and population growth in Kinshasa has important limitations. As elsewhere, the population growth of Kinshasa is driven primarily by natural increase (fertility) and in-migration. Regarding the former, most research on fertility in Kinshasa was conducted before the period of prolonged conflict (Bertrand, Chirhamolekwa, et al. 1990; Shapiro 1996; Shapiro and Tambashe 1994; Shapiro, and Tambashe 1997) or shortly after the conflict ended (Shapiro and Tambashe 2003), but there is relatively little research in recent years, despite the particularly rapid growth of Kinshasa during this time. Although migration is mentioned in some research on fertility in Kinshasa (Shapiro & Tambashe 2003), little is known about internal migration patterns in DRC, or in-migration to Kinshasa specifically.

Also missing is research on the relationship between migration and fertility in Kinshasa. Given that migrants are typically young, and women in sub-Saharan Africa (SSA) are most likely to migrate in their peak reproductive years (Brockerhoff and Eu 1993), migration also indirectly affects urban growth through the fertility of migrants. With stark differences in fertility between rural and urban areas in the DRC, as throughout most of SSA (Garenne 2008), migration from higher-fertility rural areas to lower-fertility urban areas has the potential to contribute to growth of the population size of urban centers like Kinshasa—*if* migration rates are substantial and migrants have higher fertility than non-migrants.

With the vast majority of urban population growth in the coming decades taking place in cities of the developing world (United Nations 2012), and the important health implications of urbanization, there is a critical need for research on the factors contributing to urban growth in these settings. Here, we examine whether migrants make a disproportionate contribution to population growth in Kinshasa by examining differences in fertility profiles between in-migrants and permanent Kinshasa residents. We also identify characteristics that may influence differences in fertility by migration status. We then use detailed information on migration (duration lived in Kinshasa, number of lifetime moves, age at first migration, urban/rural classification of birthplace, and the distinction between intra-Kinshasa migration and migration to Kinshasa) to see if fertility differs by characteristics of migration.

## Backgroud

Research on the relationship between internal migration, urbanization and fertility remains limited, both in DRC and elsewhere in SSA (National Research Council 2003; White, Muhidin, et al. 2008). The lack of research on migration and fertility in the DRC and SSA contributes to a general uncertainty of how the process of urban migration and residence impacts fertility behavior (White, Muhidin, et al. 2008) and the family planning needs of migrant women (National Research Council 2003). The need for more knowledge about this topic is evident from calls for better migration study designs and more nuanced measures of migration (Montgomery et al. 2016).

Generally, urban residence has been associated with lower fertility, as explained by several theories. The higher costs of raising children in cities may dissuade some from having large families (Easterlin 1975; White, Muhidin, et al. 2008), and favorable attitudes for smaller families are more common in cities than rural areas (Cleland and Wilson 1987). Even with preferences for smaller families, individuals may be unable to limit fertility in rural areas, as family planning services are more prevalent in urban centers compared to rural areas of SSA (Cleland, Bernstein, Ezeh et al. 2006).

However, while urban residence has long been associated with lower fertility in SSA (Shapiro and Tambashe. 2002), the relationship between fertility and migration to and subsequent residence in an urban area is less clear. The limited existing research on this topic has found mixed results. Most cross-sectional studies have found that migration to urban areas is associated with lower fertility (Brockerhoff 1995; Brockerhoff 1998; Omondi and Ayiemba 2005), both for migrants themselves (Brockerhoff and Yang 1994) and for subsequent generations born in the urban place of destination (White et al. 2005). Similarly, two recent longitudinal studies of migration and fertility in Ghana found lower fertility among rural-to-urban migrants (Chattopadhyay, White, Debpuur, et al. 2006; White, Muhidin, et al. 2008) compared to rural non-residents. Some older studies in SSA, however, found no fertility decline with urban migration or a positive relationship between fertility and migration (Lee 1992; Cleveland 1991).

Similarly, theories on the relationship between urban migration and fertility suggest that migrants could have either higher or lower fertility than non-migrants. Four competing theoretical approaches have been used to explain the relationship between internal migration and fertility: 1) selection 2) adaptation, 3) socialization, and 4) disruption. Regarding selection, migrants are a self-selected group for whom relatively lower fertility preferences may be part of the motivation to move to a new area (Kulu 2005; White, Muhidin, et al. 2008). Migrants differ from non-migrants in key characteristics even before migrating, some of which may be associated with both migration and lower fertility (e.g., higher education levels and aspirations, social mobility desires, higher socio-economic status) (White et al. 2005). Thus, women with profiles or desires similar to those at the place of urban destination—including for education, fertility preferences, willingness to use modern methods—may have had lower fertility outcomes regardless of whether they migrated (Rokicki, Montana, and Fink 2014). Similarly, the disruption hypothesis also implies lower fertility among migrants: migrants’ fertility behavior will be low in the period immediately prior to or following a residential change, due to the disruption in economic and social support that often results from a move (Kulu 2005).

On the other hand, migrants may have higher fertility than non-migrants. The socialization hypothesis suggests that the fertility of migrants will primarily reflect fertility preferences dominant in their place of origin, with the assumption that norms about fertility are learned at a young age, and any changes in fertility behavior among migrants will only occur over the longer-term (Kulu 2005; White et al. 2005). If migrant areas of origin are typically rural, rural-to-urban migrants may therefore have higher fertility than non-migrants at the place of destination (Kulu 2005). Alternatively, under the adaptation hypothesis, migrant fertility may eventually come to resemble the dominant patterns of the destination location, but only after higher fertility preferences among migrants erode with relatively long durations of residence with different fertility norms from origin areas (Lee 1992; Rokicki, Montana, and Fink 2014; White, Muhidin, et al. 2008). In SSA, this adaptation generally assumes that desired family size will decrease following internal migration to urban areas, due in part to the increased acceptance of and access to contraception and abortion in urban areas (Shapiro and Tambashe. 2002).

The reason for moving to an urban area may also provide insight into the relationship between fertility and migration. Some reasons for internal urban migration involve characteristics that are typically associated with lower fertility, like attending school or starting a job (Romaniuk 2011; Shapiro 1996). Other research has shown that moving to begin a marriage is common in some parts of sub-Saharan Africa (Anglewicz 2012, Reniers 2003), which suggests that fertility may increase as a result of migration. Research in SSA has often found higher fertility and frequent marriage-related migration among women in virilocal and patrilineal societies (Anglewicz 2012, Mitchell 1961).

Much remains unknown about the migration/fertility association in most of SSA, due largely to limited data on migration. Migration differs by destination and origin (e.g., urban, rural), distance travelled, permanence, duration, and motivation, among other characteristics. Yet the demographic literature on fertility and migration has often neglected to account for variation in migration characteristics in developing countries and often uses a simple binary measure of migrant/non-migrant (e.g., Brockerhoff and Yang 1994; Chattopadhyay, White, Debpuur, et al. 2006; Omondi and Ayiemba 2005; Oucho and Gould 1993). Although different migration streams (urban-rural, rural-urban, urban-urban, rural-rural, etc.), the duration of time at destination and age at migration have been previously examined on rare occasions (Brockerhoff and Yang 1994, Chattopadhyay, White, Debpuur, et al. 2006; Corker 2015) they have not been consistently studied enough to draw conclusions. Recent research has called for greater detail of migration patterns, a valid concern (Deane et al. 2010; Montgomery et al. 2016), but one that is often difficult to respond to due to continued data limitations.

We take advantage of detailed migration information to examine the association between fertility and particular characteristics of migration. Specifically, we investigate the association between fertility and (1) duration of residence in Kinshasa, (2) number of lifetime moves, (3) age at first migration, (4) urban/rural classification of birthplace, and (5) the distinction between intra-Kinshasa migration and migration to Kinshasa. Using these measures, this research expands on previous analyses of migration and fertility. It is important to note that we do not intend to estimate the effect of migration on fertility in this research. Instead, we aim to identify differences in fertility between migrants and non-migrants in Kinshasa, and explore possible explanations for these differences.

## Data and methods

### Setting

The DRC is Africa’s third most-populous country, with an estimated population of just over 77 million people according to UN estimates for 2015 (United Nations 2015a). The DRC is also one of the region's fastest growing countries and is projected by the UN to be one of the 10 most populous countries in the world by 2050. The DRC is consistently among the very poorest countries in the world, and ranked 186 out of 187 countries in the 2014 Human Development Index (United Nations 2015b). Given its country profile, it is not surprising that the DRC has one of the highest fertility rates in the world: the most recent Demographic and Health Survey (DHS) from 2013-14 estimated a country-level TFR of 6.6, a slight increase since the 6.3 TFR estimated from the 2007 DHS (Ministère du Plan et Macro International 2008; Ministère du Plan et Suivi de la Mise en oeuvre de la Révolution de la Modernité (MPSMRM), Ministère de la Santé Publique (MSP) et ICF International (2014)).

The sharp fertility differential between Kinshasa and the rest of the DRC, including other urban areas, makes it an ideal context in which to examine the relationship between migration and urban residence with fertility outcomes. Since the 1960s, fertility has remained stubbornly high throughout the DRC, although Kinshasa is a notable exception (Romaniuk 2011), the capital has a TFR of 4.2, considerably lower than other urban (5.4) and rural (7.3) areas of the DRC (MPSMRM, MSP and ICF International 2014). The modern contraceptive prevalence rate (MCPR) is extremely low throughout the DRC, at just 7.8% for the country as a whole, with condom use accounting for half of the MCPR (MPSMRM, MSP & ICF International 2014). In Kinshasa, the MCPR in 2013–14 was 19%, making it more than double the national average but still among the lowest of all capital cities in SSA. Traditional method use, on the other hand, is comparatively high in DRC and generally exceeds the MCPR, including in Kinshasa, where 25.7% of women resort to traditional methods compared to 12.6% at the national level. The DRC is predominantly virilocal and patrilineal, but there is variation in kinship patterns across ethnic groups and geographic regions (Romaniuk 2011).

### Data

Our data comes from Performance, Monitoring, and Accountability 2020 project (PMA2020). PMA2020 currently operates in eleven countries worldwide and was established in part to measure uptake of contraceptive use in many of the world’s most populous countries (http://www.pma2020.org/). To achieve this aim, PMA2020 collects representative data in these countries on an annual basis for a range of fertility and family planning-related measures. This study has received approval to collect data from Institutional Review Boards at Johns Hopkins University, Tulane University, and the University of Kinshasa. PMA2020 was supported by the Bill and Melinda Gates Foundation, Seattle, WA; under grant # OPP1079004.

We use the first round of data from Kinshasa, DRC under PMA2020, collected from November 2013 to January 2014. The sampling approach and was designed to obtain a sample that is representative of Kinshasa, and to be comparable to the DHS data for Kinshasa. The sampling framework uses a two-stage cluster sampling approach, in which the study first randomly selected census enumeration areas within Kinshasa, then conducted a listing of households in these EAs, and randomly selected 33 households within each EA. All resident women of reproductive ages within the household were selected for interview. The PMA2020 survey included basic demographic information, and information on fertility preferences and contraceptive use. PMA2020 also asked women to report the number of lifetime births, the timing (month and year) of their two most recent births, and child mortality. Because we do not have complete birth histories, we cannot establish the order of events between all births and moves, and our analysis therefore does not seek to measure the effect of migration on fertility.

A goal of this research is to address some of the limitations in common measures of migration, such as those collected by DHS. The DHS asks two questions about migration: “How long have you been living continuously in (NAME OF CURRENT PLACE OF RESIDENCE)?” and “Just before you moved here, did you live in a city, in a town, or in a rural area?” As suggested by theories on the relationship between internal migration and fertility, there are important features of the migration process that are associated with fertility but not captured by these questions. While someone may have moved from another urban area in the most recent migration, they may originally be from a rural area, and the first move from rural to urban, for example, would therefore be missed by the DHS questions. This is important in the context of the “socialization” hypothesis, which suggests that migrants are influenced by the fertility norms of their birthplace. Under the adaption theory, the fertility norms of migrants may eventually conform to those of the destination with longer durations at destinations and away from areas of origin. As a result, the age at first migration and duration at current residence may be associated with fertility. DHS collects information only on the latter. In the DHS survey, the household is considered the “current place of residence”. As a result, it is not possible to distinguish between migrants who moved within Kinshasa from those that moved to Kinshasa from another urban area of DRC in the DHS data. Since Kinshasa has lower fertility than all other provinces in DRC, one would expect that intra-Kinshasa migrants do not have higher fertility than Kinshasa non-migrants, but those moving to Kinshasa may have higher fertility than both. Since theory suggests that migration may disrupt fertility, the number of lifetime moves is relevant to fertility. Finally, it is important to note that, the relatively few questions on migration from the DHS survey are not included in every round or country; the DHS Phase 6 survey, the most recent DHS in DRC (2013–14), did not include these questions.

The PMA2020 data offer several important advantages over migration data collected by DHS. The first round of the PMA2020 study in Kinshasa included an extensive residence/migration history, which contains information on all locations where respondents previously lived for 6 months or more, and selected characteristics of these former residences. The survey captures the year and month in which the respondent moved to a particular area, along with reasons for the migration, and urban or rural classification for each place of residence. We use this information to categorize migrants as women who moved to Kinshasa from another location (women who were born in and later moved within Kinshasa are not considered migrants). Using the PMA2020 residence histories, we are able to create several characteristics of migration, including the duration lived in Kinshasa, number of lifetime moves, age at first migration, urban/rural classification for birthplace, and the distinction between intra-Kinshasa migration and migration to Kinshasa.

### Analytic methods

We begin by tabulating background characteristics for our study population, including age, marital status, level of education, number of lifetime births, and household wealth. Household wealth is measured using a constructed wealth index based on ownership of 25 household durable assets, house and roof material, livestock ownership and water source. Here again, PMA2020 uses measures similar to those in the most recent DRC DHS survey. A wealth index was created using principal component analysis (Filmer & Pritchett 2001), which is then converted into quintiles. We also focus on several measures of fertility desires and family planning, including current contraceptive use (modern and traditional), unmet need, desire to not have another child (using the question “Would you like to have a/another child or would you prefer not to have any/any more children?”), and whether the last birth was unintended (“At the time you became pregnant, did you want to become pregnant then, did you want to wait until later, or did you not want to have any/any more children at all?”). We then test for statistically significant differences in these characteristics between migrants and non-migrants.

Next, we utilize the migration histories to categorize several characteristics of migration, many of which are not included in studies of migration and fertility. We tabulate the number of lifetime moves among our respondents, the duration of time spent living in Kinshasa, migration stream (rural-urban, urban-urban), and the age at migration to Kinshasa.

We then use multivariate Poisson regressions with the number of cumulative births at the time of the survey as the dependent variable. The main independent variable of interest is the binary category of migration, compared to non-migrants. To explore whether any fertility differences among migrants are related to differences in age, education or other background characteristics, we also control for characteristics that significantly differ by migration status and likely affect fertility, specifically: age, level of education, marital status, and household wealth. Next, to examine whether differences in fertility between migrants and non-migrants are due to differences in contraceptive use between these groups, we add a measure of contraceptive use to this model, measured as a three-category variable for (0) not using any contraceptive method, (1) using a traditional contraceptive method, and (2) using a modern contraceptive method.

We next measure differences in family planning and fertility preferences by migration status by running three logistic regressions where the dependent variables are overall contraceptive use, traditional contraceptive use, and modern contraceptive use. Then, we examine fertility-related attitudes, specifically unmet need for family planning, desire to have another child, and whether the most recent birth was unintended. We run separate logistic regressions to examine whether these measures differ by migration status.

Finally, we examine whether fertility differs by particular characteristics of migration, specifically (1) the duration of time living in Kinshasa, (2) the number of lifetime moves, (3) whether their birthplace was urban or rural, (4) their age at first migration, and (5) a distinction between migration within Kinshasa and migration to Kinshasa. Number of lifetime moves is measured as categorical, with those moving from one, two, and three or more times compared to the reference group of non-migrants. Duration of time in Kinshasa is categorical, according to short-term, medium-term and longer-term residence in Kinshasa: 0–5 years, 6–20 years, and 21 or more years resided in Kinshasa. Age at first migration is measured in 10-year age intervals. Alternative categorizations yielded results that were not substantively different. The same regression controls from previous models are included here.

## Results

Table 1 shows background characteristics for migrants and non-migrants. Of the 2197 women in the sample, 340 (15.5%) are classified as migrants. Several significant socioeconomic differences between migrants and non-migrants are evident in Table 1. Most notably, migrants are older and more likely to be married or in union than non-migrants. Somewhat surprisingly, migrants and non-migrants have nearly identical levels of education: the only significant difference for no education, 8.5% of migrants compared to 5.4% of non-migrants, with all other categories similar in proportion and not statistically significantly different. No significant differences were found for wealth status between migrants and non-migrants.

**Table 1:** Background characteristics for migrant and non-migrant women, PMA2020 Project, Kinshasa 2013

	Non-migrants	Migrants
Mean age (SD)	26.8 (0.2)	30.6** (0.5)
Level of education		
No education	5.4%	8.5%*
Primary school	47.1%	47.1%
Middle secondary	39.7%	37.7%
Advanced secondary +	7.8%	6.7%
Marital status		
Married/in union	47.3%	64.1%**
Separated/divorced/widowed	3.3%	5.0%
Never married	49.4%	30.9%**
Household wealth		
Quintile 1 (lowest)	19.7%	17.9%
Quintile 2	20.1%	21.8%
Quintile 3	20.3%	20.9%
Quintile 4	20.2%	20.0%
Quintile 5 (highest)	19.7%	19.4%
Fertility-related characteristics		
Total fertility rate (TFR)	4.00	4.76**
Contraceptive use (any method)	32.3%	35.0%
Traditional contraceptive use	15.9%	22.1%**
Modern contraceptive use	16.1%	12.9%
Unmet need for contraception	16.5%	24.1%**
Do not want another child	26.7%	39.2%**
Most recent birth unintended	54.2%	54.3%
N=	1,857	340

Difference between migrants and non-migrants significant at *p ≤ 0.10; **p ≤0.05; ***p ≤ 0.01. Unmet need was calculated only for ever-married women (*n* = 1175), and recent birth unintended was available only for women ever giving birth (*n* = 1296)

Characteristics related to family planning and fertility preferences show greater differences between the two groups. Although levels of contraceptive use are similar (32.3% for migrants and 35.0% for non-migrants), traditional method use is higher and modern method use is lower among migrants. While nearly a quarter of migrant women (24.1%) have an unmet need for contraception, only 16.5% of non-migrants do. Notably, 39.2% of migrants say they want no more children while only 26.7% of non-migrants expressed the same desire.

According to migration characteristics (Table 2), most migrants moved more than once in their lifetime, with an average of 2.0 lifetime moves among migrants. As with migration patterns elsewhere, the majority of women moved during young adulthood: the average age at migration to Kinshasa was 18.1 years old, and nearly 70% of migrants moved between ages 10 and 30. Most migrants in our sample had been residing in Kinshasa for over a decade: the average duration spent in Kinshasa is 12.6 years. Migrant women were slightly more likely to have previously lived in an urban (8.4% of all women, 54.1% of migrants) than rural area (7.1% total, 45.9% of migrants). International in-migration is uncommon: of the 340 migrants, only 7 had moved to Kinshasa from another country (five from the Republic of the Congo and two from Rwanda).

**Table 2:** Migration characteristics, PMA2020 Project, Kinshasa 2013

Mean number of moves (SD)	2.0 (0.03)
Resided in Kinshasa for	
0–9 years	49.7%
10–19 years	26.8%
20–29 years	12.3%
30+ years	11.2%
Migration stream	
Rural-to-Kinshasa migrant	45.9%
Urban-to-Kinshasa migrant	54.1%
Age at first migration	
0–9 years old	85.3%
10–19 years old	8.2%
20+ years old	6.5%
*N*=	340

We find that fertility is significantly higher for migrants compared to non-migrants in Kinshasa. Migrant women had on average cumulative fertility of at the time of the survey of 4.76 children compared to 4.00 for non-migrants in Kinshasa. However, the difference in cumulative fertility between migrants and non-migrants is relatively small and perhaps less than would be expected given the much higher fertility from the migrants' places of origin: the TFR in other (non-Kinshasa) urban areas of DRC is 6.03, and is 7.25 in rural DRC (using DHS data using two-year ASFRs) (MPSMRM, MSP and ICF International, 2014), making a difference of less than one child per woman seem small by comparison.

The fertility differences between migrants and non-migrants in Kinshasa, however, are nonetheless significant, leading us to ask: is the higher fertility among migrants due to the differences in age, education or other background characteristics? Results shown in Table 3 (model 1) suggest that even after controlling for differences in background characteristics (age, education, marital status, household wealth), migrants have slightly but significantly higher cumulative births when individual-level characteristics are held constant. Our second model in Table 3 shows that contraceptive use partly explains the higher fertility among migrants. After controlling for differences in the use of contraceptive methods between migrants and non-migrants (model 2), we find that the measure for migrant declines in the level of statistical significance and effect size. Differences in contraceptive use do not, however, do not completely explain differences in fertility between migrants and non-migrants, as the measure of migration is still statistically significant (at *p* = 0.051).

**Table 3:** Poisson regression results for differences in lifetime fertility between migrants and non-migrants, PMA2020 Project, Kinshasa 2013

	Model 1		Model 2	
	Coef	SE	Coef	SE
Migrant	0.08**	*0.04*	0.07*	*0.04*
Age	0.27***	0.02	0.26***	0.02
Age^2^	−0.01***	0.00	−0.00***	0.00
Level of education				
No education (ref.)	----	----	----	----
Primary	−0.10*	0.05	−0.10*	0.05
Middle secondary school	−0.36***	0.06	−0.35***	0.06
Advanced secondary or higher	−0.71***	0.09	−0.72***	0.09
Marital status				
Married/partnered (ref.)	----	----	----	----
Separated/divorced/widowed	−0.21***	0.06	−0.16***	0.07
Never married	−0.98***	0.06	−0.98***	0.06
Household wealth				
Quintile 1 (lowest, ref.)	----	----	----	----
Quintile 2	−0.03	0.05	−0.04	0.05
Quintile 3	−0.04	0.05	−0.05	0.06
Quintile 4	−0.17***	0.05	−0.18***	0.06
Quintile 5 (highest)	−0.18***	0.06	−0.21***	0.06
Contraceptive use				
Not using contraception (ref.)			----	----
Using traditional contraception			0.14***	0.04
Using modern contraception			0.21***	0.04
*N*=	2197		2197	

*p ≤ .10; **p ≤ .05; ***p ≤ .01

Results for fertility preferences and family planning-related characteristics may yield some insight into the differences in fertility between migrants and non-migrants. First, we find no significant differences in overall contraceptive use between migrants and non-migrants (Table 4). When this is disaggregated into contraceptive method type, however, we find that migrant women are significantly less likely to use modern contraception and significantly more likely to be using traditional methods compared to Kinshasa natives. Second, the difference in fertility does not appear to be related to greater preferences for limiting, as we see no evidence of a significant difference between migrants and non-migrants with regard to the desire to not have another child (Table 5). Similarly, there are no significant differences between migrants and non-migrants in unmet need or whether a woman's last birth was unintended.

**Table 4:** Logistic regression results for differences in current contraceptive use between migrants and non-migrants, PMA2020 Project, Kinshasa 2013

	Using any method of contraception		Modern contraception		Traditional contraception	
	Odds	SE	Odds	SE	Odds	SE
Migrant	*0.95*	*0.13*	0.70**	*0.13*	1.28*	*0.20*
Number of births	1.21***	0.05	1.22***	0.06	1.09**	0.05
Age	1.38***	0.06	1.26***	0.07	1.33***	0.07
Age^2^	0.99***	0.00	0.99***	0.00	0.99***	0.00
Level of education						
No education (ref.)	----	----	----	----	----	----
Primary school	0.97	0.23	1.11	0.34	0.88	0.23
Middle secondary	1.30	0.31	1.91**	0.60	0.83	0.23
Advanced secondary +	2.13**	0.63	2.56**	0.94	1.24	0.42
Marital status						
Married/in union (ref.)	----	----	----	----	----	----
Separated/divorced/widowed	0.22***	0.08	0.30**	0.16	0.27***	0.13
Never married	0.98	0.13	0.99	0.17	0.98	0.16
Household wealth						
Quintile 1 (lowest, ref.)	----	----	----	----	----	----
Quintile 2	1.13	0.19	0.84	0.18	1.37	0.28
Quintile 3	1.40**	0.23	0.88	0.18	1.82***	0.37
Quintile 4	1.23	0.21	1.05	0.22	1.29	0.28
Quintile 5 (highest)	1.51**	0.27	1.26	0.27	1.41	0.31
*N*=	2010		2010		2010	

Notes: *p ≤ 0.10; **p ≤ 0.05; ***p ≤ 0.01. We remove all pregnant women from this analysis (*n* = 187, 160 non-migrants and 27 migrants)

**Table 5:** Logistic regression results for differences in fertility preferences between migrants and non-migrants, PMA2020 Project, Kinshasa 2013

	Unmet need		Want another child		Most recent birth unintended	
	Odds	SE	Odds	SE	Odds	SE
Migrant	*1.11*	*0.18*	*1.01*	*0.18*	*1.07*	*0.17*
Births	1.12***	0.04	2.21***	0.13	1.49***	0.07
Age	0.95***	0.01	0.64***	0.04	0.72***	0.05
Age^2^	----	----	1.01***	0.00	1.00***	0.00
Level of education						
No education (ref.)	----	----	----	----	----	----
Primary school	1.49	0.37	1.28	0.37	1.42	0.33
Middle secondary	1.16	0.31	1.12	0.34	1.56*	0.40
Advanced secondary +	0.57	0.22	1.46	0.60	0.84	0.31
Marital status						
Married (ref.)	----	----	----	----	----	----
Separated/divorced/widowed	2.99***	0.77	1.74	0.62	1.22	0.34
Never married	----	----	1.29	0.25	4.09***	0.78
Household wealth						
Quintile 1 (lowest, ref.)	----	----	----	----	----	----
Quintile 2	1.12	0.21	0.91	0.19	1.43*	0.28
Quintile 3	0.99	0.20	1.09	0.23	0.91	0.18
Quintile 4	0.81	0.17	1.10	0.24	0.94	0.20
Quintile 5 (highest)	1.15	0.26	1.12	0.25	1.09	0.25
*N*=	1175		2197		1296	

*p ≤ 0.10; **p ≤ 0.05; ***p ≤ 0.01; the quadratic term for age was not statistically significant in the model for unmet need and was therefore left out; unmet need was calculated only for ever-married women, and recent birth unintended was available only for women ever giving birth

Finally, our results indicate fertility is associated with several characteristics of migration. As shown in the first model in Table 6, we find that longer duration in Kinshasa is associated with higher fertility: migrants who had lived in Kinshasa for 21 or more years had 0.12 more children than permanent Kinshasa residents, while there are no significant differences compared to Kinshasa natives for women who migrated within the past 20 years. Similarly, we see a positive relationship between number of lifetime moves and cumulative fertility (Table 6, model 2). While there are no significant differences in fertility between non-migrants and migrants born in rural areas, we find significantly higher fertility for migrants born in other urban areas who later moved to Kinshasa compared to non-migrants, shown in Table 6, model 3. We also performed similar analysis for urban/rural classification of the most recent previous residence (results not shown), and results also showed that urban-to-Kinshasa migrants had significantly higher fertility, but the level of statistical significance was lower (*p* < 0.10) and the coefficient was smaller (0.09) than results for birthplace. We also find statistically significant differences by age at first migration, in which women who first moved by age 20 had significantly higher fertility than women who did not move (Table 7, model 4). In results not shown, there were no significant differences in fertility by age at most recent migration. Finally, we find an important distinction between individuals who move within Kinshasa, and those who move into Kinshasa from elsewhere. While those moving to Kinshasa from outside have significantly higher fertility, individuals who move within Kinshasa do not (Table 7, Model 5).

**Table 6:** Poisson regression results for the relationship between migration characteristics and fertility, PMA2020 Project, Kinshasa 2013

	Model 1			Model 2			Model 3	
	Coef.	SE		Coef.	SE		Coef.	SE
Duration in Kinshasa			Number of moves			Migration stream ^a^		
Born in Kinshasa (ref.)	----	----	No moves/non-migrant (ref.)	----	----	Non-migrant (ref.)	----	----
0–5 years in Kinshasa	0.11	0.07	One move	−0.01	0.05	Rural-Kinshasa migrant	0.05	0.05
6-20 years in Kinshasa	0.00	0.06	Two moves	0.07*	0.04	Urban–Kinshasa migrant	0.10**	0.05
21+ years in Kinshasa	0.12**	0.06	Three + moves	0.17**	0.09			
Age	0.27***	0.02	Age	0.27***	0.02	Age	0.27***	0.02
Age^2^	−0.01***	0.00	Age^2^	−0.00***	0.00	Age^2^	−0.00***	0.00
Level of education			Level of education			Level of education		
No education (ref.)	----	----	No education (ref.)	----	----	No education (ref.)	----	----
Primary school	−0.10*	0.05	Primary school	−0.10*	0.05	Primary school	−0.10*	0.05
Middle secondary	−0.36***	0.06	Middle secondary	−0.35***	0.06	Middle secondary	−0.36***	0.06
Advanced secondary +	−0.71***	0.09	Advanced secondary +	−0.71***	0.09	Advanced secondary +	−0.71***	0.09
Marital status			Marital status			Marital status		
Married (ref.)	----	----	Married (ref.)	----	----	Married (ref.)	----	----
Sep/div/wid	−0.21***	0.06	Sep/div/wid	−0.21***	0.06	Sep/div/wid	−0.21***	0.06
Never married	−0.98***	0.06	Never married	−0.98***	0.06	Never married	−0.98***	0.06
Household wealth			Household wealth			Household wealth		
Quintile 1 (lowest, ref.)	----	----	Quintile 1 (lowest, ref.)	----	----	Quintile 1 (lowest, ref.)	----	----
Quintile 2	−0.03	0.05	Quintile 2	−0.04	0.05	Quintile 2	−0.03	0.05
Quintile 3	−0.04	0.05	Quintile 3	−0.04	0.05	Quintile 3	−0.04	0.05
Quintile 4	−0.17***	0.05	Quintile 4	−0.17***	0.05	Quintile 4	−0.17***	0.05
Quintile 5 (highest)	−0.19***	0.06	Quintile 5 (highest)	−0.18***	0.06	Quintile 5 (highest)	−0.18***	0.06
*N*=	2197		*N*=	2197		*N*=	2197	

*p ≤ 0.10; **p ≤ 0.05; ***p ≤ 0.01; ^a^=“Migration stream” is measured as the urban/rural classification of the migrant’s birthplace

**Table 7:** Poisson regression results for the relationship between migration characteristics and fertility, PMA2020 Project, Kinshasa 2013

	Model 4			Model 5	
	Coef.	SE		Coef.	SE
Age at first migration			Intra- and outside-Kinshasa migration		
Non-migrant (ref.) ----		----	Non-migrant (ref.)	----	----
0–9 years old	0.05	0.04	Intra-Kinshasa migrant	0.00	0.06
10–19 years old	0.09	0.15	Migrant to Kinshasa	0.08**	0.04
20+ years old	0.28***	0.14			
Age	0.27***	0.02	Age	0.27***	0.02
Age^2^	–0.00***	0.00	Age^2^	-0.00***	0.00
Level of education			Level of education		
No education (ref.)	----	----	No education (ref.)	----	----
Primary school	−0.10*	0.05	Primary school	−0.10*	0.05
Middle secondary	−0.36***	0.06	Middle secondary	−0.36***	0.06
Advanced secondary +	−0.72***	0.09	Advanced secondary +	−0.71***	0.09
Marital status			Marital status		
Married (ref.)	----	----	Married (ref.)	----	----
Sep/div/wid	−0.21***	0.06	Sep/div/wid	−0.21***	0.06
Never married	−0.98***	0.06	Never married	−0.98***	0.06
Household wealth			Household wealth		
Quintile 1 (lowest, ref.)	----	----	Quintile 1 (lowest, ref.)	----	----
Quintile 2	−0.03	0.05	Quintile 2	−0.03	0.05
Quintile 3	−0.04	0.05	Quintile 3	−0.04	0.05
Quintile 4	−0.17***	0.05	Quintile 4	−0.17***	0.05
Quintile 5 (highest)	−0.19***	0.06	Quintile 5 (highest)	−0.18***	0.06
*N*=	2197		*N*=	2197	

*p ≤ 0.10; **p ≤ 0.05; ***p ≤ 0.01

## Discussion

We find that migrants have significantly higher fertility than permanent Kinshasa residents. The higher fertility for migrants remains even after controlling for characteristics that differ from non-migrants, suggesting that even though migrants systematically differ from non-migrants with regard to age and marital status, differences in fertility are not explained by differences in background characteristics. Our results therefore suggest that migrants contribute to the population growth of Kinshasa both directly (via migration) and indirectly (through their relatively higher fertility).

However, the difference in fertility between migrants and non-migrants is relatively small in magnitude when considering the difference in fertility levels between Kinshasa and the rest of the country. Our results show a TFR for migrants is 4.76, compared to 4.00 for non-migrants, compared to a TFR in other (non-Kinshasa) urban areas of DRC of 6.03, and 7.25 in rural DRC (with DHS data using 2-year ASFRs) (MPSMRM, MSP & ICF International, 2014). In other words, the TFR for migrants to Kinshasa is much closer to permanent Kinshasa residents than areas of origin.

Based on analysis of family planning-related outcomes, it appears that the higher fertility of migrants could be due to their greater use of less effective contraception: migrants are more likely to use traditional methods and less likely to use modern contraceptive methods, and the difference in fertility between migrants and non-migrants declines in effect size and statistical significance once we control for contraceptive use. The higher rate of traditional method use among migrants could be a preference, or due to from barriers to obtaining modern methods: although there were no significant differences in knowledge of a location to obtain family planning methods (results not shown), migrants may face more barriers to accessing family planning services (Irani et al. 2013; Shapiro and Tambashe 1994), suggesting that many migrants may turn to traditional methods in the absence of available modern methods. This finding is in line with previous research on contraceptive use and migration in Kinshasa, which also found that migration status was associated with a decreased likelihood of using modern methods, but not for using any method of contraception (Shapiro and Tambashe 1994), suggesting that a higher likelihood of traditional than modern method use by migrants in Kinshasa is consistent over time.

The more-detailed measures of migration contribute to our knowledge on the association between migration and fertility in Kinshasa. Perhaps surprisingly, cumulative fertility is higher among migrants who have lived longest in Kinshasa, even after controlling for age. We also find that migration does not appear to lower fertility due to disruption: more lifetime moves is associated with higher fertility in our sample, which could potentially instead reflect the disruptive effect of migration on access to family planning. We find that the higher fertility among migrants in our sample appears to be driven primarily by urban-Kinshasa migrants, who have significantly higher fertility than non-migrants, while no difference is found for rural migrants into Kinshasa. This may represent different patterns of adaptation between these two groups or stronger selection those seeking higher education from rural areas. It could also result from different age patterns fertility and different patterns of return migration of these two groups, if for example a greater proportion of women migrate at younger ages from rural areas (for schooling or work) and have low fertility while in Kinshasa but are also more likely to return to their villages during peak childbearing years while migrants from urban areas are more likely to stay in Kinshasa permanently. Higher fertility among migrants is concentrated among those who first migrated at relatively older ages (by age 20), which provides some evidence for the socialization hypothesis. Finally, we show that the distinction between intra-Kinshasa migration and moving to Kinshasa is important, as migrants to Kinshasa have significantly higher fertility than non-migrants, but intra-Kinshasa migrants are not different. It is important to note that the order of events between migration and fertility cannot be accurately measured with this dataset (such data are rare in any setting, particularly SSA), but our nuanced measures, which are also seldom available, nonetheless yield insight into migration processes in SSA.

We also tested other possible explanations for higher fertility among migrants for which no significant associations were found. There did not appear to be differences in geographic access to family planning: migrants were no different from non-migrants in knowledge of a location to obtain family planning methods, although it is possible they may face other barriers to access. Similarly, as mentioned above, there was no statistically significant relationship between age at most recent migration and cumulative fertility.

Lower fertility among urban migrants compared to non-migrants in the places of origin has been found in much of the recent literature on SSA (Brockerhoff 1998; Omondi and Ayiemba 2005; White, Muhidin, et al. 2008; White et al. 2005). This is also closely aligned with recent studies from West Africa on internal migration to a capital city, which also found remarkably similar fertility outcomes between migrants and non-migrants in Accra, Ghana (Rokicki, Montana, and Fink 2014). Prior research suggests an increased use of modern contraception after urban migration impacts lower fertility (Brockerhoff and Eu 1993), while this may be the case for Kinshasa migrants as well, it appears that the marginally higher fertility may be partly explained by greater reliance on traditional methods. Earlier research in Kinshasa also found that migrant women were less likely to report having had an abortion, suggesting higher abortion rates among Kinshasa natives may also play a role in their lower fertility outcomes (Shapiro and Tambashe 2003).

There are several possible explanations for our results. Our results are generally compatible with the selection hypothesis, that migration may select individuals with differing fertility profiles. We suspect this selection effect based largely on the similar education profiles of migrants and non-migrants, which was particularly surprising given the differences in average years of education among women in Kinshasa (10.4), compared to other urban areas of the DRC (8.0) and rural DRC (4.5) (MPSMRM, MSP and ICF International, 2014). As few other cities in Kinshasa have universities and many rural areas have only primary schools, in order to attain the similar education profile of non-migrants, most migrants either had relatively high educational attainment at their places of origin prior to moving (unlikely, if not impossible, in most rural areas) or migrated to Kinshasa in order to increase their educational attainment—both scenarios which imply selection in terms of education. The similar fertility between migrant and non-migrants also hints at adaptation following migration (although we cannot distinguish clearly between fertility prior to and following migration), particularly as migrant rates of contraceptive use, while they lag behind those of non-migrants, are much higher than for women in their places or origin, suggesting changes in their contraceptive practices following relocation in Kinshasa. In addition, there is some evidence for socialization, as individuals who first migrate at older ages have higher fertility than Kinshasa non-migrants (although their fertility is still lower than most other rural or urban areas of DRC).

In contrast, we do not find support for other possible reasons for differential fertility among migrants. For example, we do not find that migrants maintain fertility closer to that found in their place of origin, given that the TFR for migrants is closer to permanent Kinshasa residents than areas of origin (as described above). In addition, we do not find evidence of adaptation; fertility does not decline with greater durations of time in Kinshasa; in fact we find that fertility is highest among longer-term migrants. In addition, migration does not appear to disrupt fertility, as we also find higher fertility with more moves.

We do not intend to estimate the effect of migration on fertility in this research. Instead, we seek to identify differences in fertility between migrants and non-migrants in Kinshasa, and explore possible explanations for these differences. Establishing a causal relationship between migration and fertility would require longitudinal data that includes complete birth histories and precise timing of births and migration—a rarity for SSA. Without complete birth histories for respondents, we cannot identify the order of events between births and migration and thus cannot determine whether the higher fertility found for migrants is a result of higher fertility prior to migration or higher levels of fertility by migrants after re-locating to Kinshasa. Similarly, without longitudinal data, it is not possible to establish the order of events; for example, was economic status affected by migration to Kinshasa, and/or did migration select individuals of differing economic status? In addition, the survey did not measure some factors that may be associated with fertility and differ by migration status, such as employment, which research in Kinshasa prior to the period of conflict found to be highly correlated with contraceptive use and access to abortion (Shapiro and Tambashe 2003). Although one of the main advantages of the PMA2020 Kinshasa dataset is its comparability to the DHS data for Kinshasa, because the 2014 DRC DHS did not include variables on migration (as these were dropped from the DHS Round 6 core questionnaire in most countries), we are unable to compare our results with the DRC DHS (although our fertility estimates for Kinshasa as whole match closely those from the DHS).

Even without identifying the causal effect of migration on fertility and related outcomes, these findings nonetheless suggests there are important differences in migrant fertility and family planning in Kinshasa that could be considered in current family planning program approaches and for more detailed study in further research. Most notably, the higher use of traditional methods by migrants may signal a greater unmet need for modern contraception by this group. While the higher traditional method use by migrants could likewise signal preference for these methods, we think this is unlikely given that traditional method use is higher in Kinshasa than in other urban areas or rural areas (17.4% in Kinshasa compared with 10.0% in other urban areas and 8.9% in rural areas, according to the 2014 DHS), making it unlikely that migrants are continuing the preferred method from their places of origin. Rather, this may signal migrant adaptation to traditional methods in the absence of easy access to modern methods, meaning migrants currently using traditional methods may represent a segment of women eager to transition to modern methods. The complexity of our results also reinforces the importance of acknowledging diversity among migrants. Different hypotheses may apply for different migrant groups at different times and in different contexts, and the collection of more detailed data on both migration and birth histories is necessary to better identify dominant theories of fertility and mobility in different contexts.

## Conclusion

Our findings suggest that rather than fuel rapid increases in Kinshasa’s urban growth, continued migration to Kinshasa may also have a positive contribution to the process of the fertility transition throughout the country, if the city continues to absorb high numbers of migrants who ultimately have significantly and substantially lower fertility than they would likely have had if they remained in their urban or rural places of origin. While more complete data on the timing of migration and fertility is needed to tease out the dominant mechanisms at play, our findings strongly suggest that residence in a new urban area may play an important role in lowering fertility rates of the substantial numbers of women who are expected to migrate to Kinshasa from other areas of the DRC in the coming years.
